# The impact of a new genus on the molecular phylogeny of Hemisphaeriini (Hemiptera, Fulgoromorpha, Issidae)

**DOI:** 10.3897/zookeys.880.36828

**Published:** 2019-10-14

**Authors:** Songping Zhao, Thierry Bourgoin, Menglin Wang

**Affiliations:** 1 Key Laboratory of Southwest China Wildlife Resources Conservation of the Ministry of Education, China West Normal University, Nanchong, Sichuan Province, 637009, China China West Normal University Nanchong China; 2 Institut de Systématique, Évolution, Biodiversité, ISYEB-UMR 7205 MNHN-CNRS-Sorbonne Université-EPHE, Muséum national d’Histoire naturelle, CP 50, 57 rue Cuvier, F-75005 Paris, France Sorbonne Université Paris France

**Keywords:** Hemisphaeriinae, molecular, morphology, new species, Oriental, planthoppers

## Abstract

A new genus, *Retaldar***gen. nov.** of the family Issidae (Hemisphaeriinae, Hemisphaeriini) is described from Guangxi Province of China. A revised molecular analysis for the Hemisphaeriini based on partial sequences of 18S, 28S, COXI and Cytb, provides evidence for a new lineage within the subtribe Mongolianina. With two subgroups of genera now identified, the monophyly of Mongolianina is discussed from both a morphological and a molecular basis.

## Introduction

Recently Wang et al. (2016) proposed important changes to the classification of the planthopper family Issidae based on the first molecular analysis of the family. Supported also by a new set of morphological characters, several new lineages were identified forming a major group including almost all Oriental genera in the subfamily Hemisphaeriinae Melichar, 1906 sec. Wang et al. (2016). This important monophyletic unit currently groups around 100 genera and 486 species distributed in the Oriental region (Bourgoin 2019) with a few taxa in the New World such as *Picumna* Stål, 1864 (Wang et al. 2016). The subfamily is divided into four tribes according to the following topology: (Kodaianellini Wang, Zhang & Bourgoin, 2016 + (Sarimini Wang, Zhang & Bourgoin, 2016 + (Parahiraciini Cheng & Yang, 1991 + Hemisphaeriini Melichar, 1906))) (Wang et al. 2016). The last tribe currently contains 28 genera and is divided into two sister subtribes: Mongolianina Wang, Zhang & Bourgoin, 2016 (6 genera) and Hemisphaeriina Melichar, 1906 (11 genera). Two unnamed lineages in each subtribe were also identified in the Wang et al. (2016) analysis. They were not discussed being considered as artificial due to sampling bias: several taxa belonged to the same genus (viz. *Mongoliona* Distant, 1909 and *Hemisphaerius* Schaum, 1850). Beside these 17 Hemisphaeriini genera, 11 other genera still remain in an *incertae sedis* position within the Hemisphaeriini (Bourgoin 2019).

In this paper, we describe and sequence the genes (18S, 28S (D3–D5) and (D6–D7), COXI and Cytb) of a new species of Hemisphaeriini from Guangxi Province of China, and provide several sequences for other known genera. These new data allow the reassessment of the subtribal division of Hemisphaeriini proposed by Wang et al. (2016).

## Materials and methods

The specimens were collected by net capture and stored in alcohol. The genitalia were separated from the insect body using micro-scissors, and then boiled in 10% NaOH solution for few minutes until muscles were completely dissolved leaving tegumentary structures. After rinsing in distilled water several times to clean the residual NaOH solution, the abdomen was subsequently transferred to glycerine for final dissection and observation. Genitalia were finally conserved under the specimen in genital vials. Photographs for external morphology and genitalia characters were taken using Leica DFC camera attached to Leica M205FA stereomicroscope and further refined with LAS X software. Morphological interpretations and subsequent terminologies follow Bourgoin (1987, 1993) for male and female genitalia respectively, Emeljanov (1971) for the term “hypocostal plate” on the forewing and Bourgoin et al. (2015) for wing venation. The type specimens are deposited in China West Normal University, Nanchong, Sichuan Province, China.

Total genomic DNA was extracted from the legs of type specimens using a Sangon Ezup column animal genomic DNA purification kit. The genes (18S, 28S, COXI, Cytb) were amplified using the same primers and amplification procedure as in Wang et al. (2016). DNA sequencing was conducted at Sangon Company (Shanghai, China). Taxon sampling includes all Hemisphaeriini taxa available from a previous analysis (Wang et al. 2016) for which several new sequences are added (Table 1). Both the newly described genus and an undescribed *Mongoliana* species from Thailand (*Mongoliana* sp. 1) were included in the new analysis. From Wang et al. (2016), 11 genera representing all major lineages were used as outgroups, plus a new species of the genus *Rhombissus*Gnezdilov & Hayashi, 2016 (*Rhombissus* sp.) from the issid tribe Parahiraciini. All sequences are registered in GenBank with their accession numbers provided in Table 1. Contigs assembly was made using the software SEQMAN from package DNASTAR v5.01 (www.dnastar.com). MEGA v7.0 (Kumar et al. 2016) was used for performing alignments. IQTREE v1.4.1 (Nguyen et al. 2015) was used for maximum likelihood phylogenetic analysis using 10,000 ultrafast bootstraps (Minh et al. 2013) with substitution model automatically selected for partitions unlinked; other parameters were used as per default. FIGTREE v1.1.2 (Rambaut 2016) was used to visualize the tree.

**Table 1.** Taxa sampling used for the phylogeny tree and corresponding sequence numbers registered in GenBank. The symbol * denotes new added sequences in this study.

Taxa name	Collecting location	Gene 18S	Gene 18S	Gene 18S	Gene 28S	Gene 28S	COXI	Cytb
		(1F–5R)	(3F–Bi)	(A2–9R)	(D3–D5)	(D6–D7)		
** Hemisphaeriini **								
*Ceratogergithus pseudotessellatus* (Che, Zhang & Wang, 2007)	China	KX761574	–	KX761576	KX761444	KX702806	KX702919	KX702906
*Ceratogergithus spinosus* (Che, Zhang & Wang, 2007)	China	KX761491	KX761491	KX761491	KX761532	KX761521	KX761502	KX761513
*Choutagus longicephalus* Zhang, Wang & Che, 2006	China	KX650620	KX650620	KX650620	KX761450	KX702810	KX761460	–
*Clypeosmilus centrodasus* Gnezdilov & Soulier-Perkins, 2017	Vietnam	–	–	KX761575	–	–	KX761470	KX761474
*Eusudasina nantouensis* Yang, 1994	China	JX196136	–	–	–	–	HM052838	HM452266
*Euxaldar lenis* Gnezdilov, Bourgoin & Wang, 2017	Vietnam	KX761573	–	KX761565	KX761412	–	–	–
*Gergithoides carinatifrons* Schumacher, 1915	China	–	KX761538	KX761538	–	KX702805	KX761555	KX702905
*Gergithoides rugulosus* (Melichar, 1906)	China	JX196163	–	–	–	–	HM052835	HM452279
*Gergithus yunnanensis* Che, Zhang & Wang, 2007	China	KX702831	KX702831	KX702831	KX761456	MN381848*	KX702924	KX702915
*Hemisphaerius palaemon* Fennah, 1978	China	KX761486	KX761486	KX761486	KX761526	KX761517	KX761497	KX761508
*Hemisphaerius rufovarius* Walker, 1858	China	KX702825	KX702825	KX702825	KX761454	KX702812	KX702923	KX702913
*Hemisphaerius lysanias* Fennah, 1978	Vietnam	KX702833	KX702833	KX702833	KX761404	KX702860	KX702933	KX702883
*Hemisphaerius coccinelloides* (Burmeister, 1834)	Philippines	KX702834	KX702834	KX702834	KX761405	KX702861	KX702934	KX702884
*Hemisphaerius* sp.	Laos	KX702835	KX702835	KX702835	KX761406	KX702862	KX761556	KX702885
*Hemisphaerius testaceus* Distant, 1906	China	JX196135	–	–	–	–	HM052831	HM452258
*Macrodaruma pertinax* Fennah, 1978	Vietnam	KX702832	KX702832	KX702832	KX761402	KX702859	KX702931	KX702882
*Macrodaruma* sp.	China	KX702828	KX702828	KX702828	KX761399	KX702857	KX702927	KX702881
*Maculergithus multipunctatus* (Che, Zhang & Wang, 2007)	China	KX702816	KX702816	KX702816	KX761443	KX702804	KX702918	KX702904
*Maculergithus nonomaculatus* (Meng & Wang, 2012)	China	KX761492	KX761492	KX761492	KX761533	KX761522	KX761503	KX761514
*Mongoliana triangularis* Che, Wang & Chou, 2003	China	–	–	KX761561	KX761528	–	–	KX761510
*Mongoliana sinuata* Che, Wang & Chou, 2003	China	KX702820	KX702820	KX702820	KX761448	–	KX761459	KX702908
*Mongoliana* sp.1	Thailand	–	–	MN422135*	MN381854*	–	–	MN332233*
*Mongoliana* sp. 2	China	KX761572	MN422136*	KX761566	KX761534	MN381849*	–	–
*Mongoliana serrata* Che, Wang & Chou, 2003	China	JX196160	–	–	–	–	HM052830	HM452272
*Neogergithoides tubercularis* Sun, Meng & Wang, 2012	China	KX702822	KX702822	KX702822	KX761451	MN381845*	KX761558	KX702910
*Ophthalmosphaerius trilobulus* (Che, Zhang & Wang, 2006)	China	KX702826	KX702826	KX702826	KX761455	KX702813	KX761462	KX702914
*Retaldar yanitubus* sp. nov.	China	MN381856*	MN381856*	MN381856*	MN381853*	MN381851*	MN381857*	MN332232*
** Thioniini **								
*Thionia* sp.	Panama		KX761539	KX761539	KX761407		KX702935	KX702886
** Hysteropterini **								
*Celyphoma quadrupla* Meng & Wang, 2012	China	KX702815	KX702815	KX702815	KX761442	KX702803	KX702917	KX702903
*Mulsantereum maculifrons* (Mulsant & Rey, 1855)	France	KX761569	KX761569	KX761569	KX761400	MN381847*	KX702928	KX761551
** Issini **								
*Issus coleoptratus* (Fabricius, 1781)	France	KX761568	KX761568	KX761568	KX761403	KX761560	KX702932	KX761550
** Kodaianellini **								
*Kodaianellissus intorqueus* Wang, Bourgoin & Zhang, 2017	China	KX761476	KX761476	KX761476	KX761480	KX761482		KX761472
*Kodaianella bicinctifrons* Fennah, 1956	China	KX702814	KX702814	KX702814	KX761441	KX702802	KX761458	KX702902
** Sarimini **								
*Sarima bifurca* Meng & Wang, 2016	China	KX702819	KX702819	KX702819	KX761447	KX702808	KX702921	KX761552
*Tetrica* sp.	China	KX702821	KX702821	KX702821	KX761449	KX702809	KX702922	KX702909
** Parahiraciini **								
*Flavina hainana* (Wang & Wang, 1999)	China	KX702824	KX702824	KX702824	KX761453	MN381846*		KX702912
*Fortunia* sp.	China	KX761487	KX761487	KX761487	KX761527	KX761518	KX761498	KX761509
*Neodurium hamatum* Wang & Wang, 2011	China	KX702818	KX702818	KX702818	KX761446	MN381844*	KX702920	
*Rhombissus* sp.	China	MN381855*	MN381855*	MN381855*	MN381852*	MN381850*		MN332231*

## Taxonomy

### Hemisphaeriini Melichar, 1906

#### Mongolianina Wang, Zhang & Bourgoin, 2016

##### Retaldar gen. nov.

Taxon classificationAnimaliaHemipteraIssidae

5FFCBB6D-BB7D-57FA-A770-F1308B65EECC

http://zoobank.org/9A9AD297-9A97-44C2-B710-B1B34A0D1817

###### Type species.

*Retaldar yanitubus* sp. nov., here designated.

###### Etymology.

Genus name masculine from the free combination of the latin word ‘*rete*’ meaning network as for the reticulated forewings and the suffix ‘-*aldar*’ from the genus *Euxaldar* Fennah, 1978.

###### Diagnosis.

**Figures 1–5.**
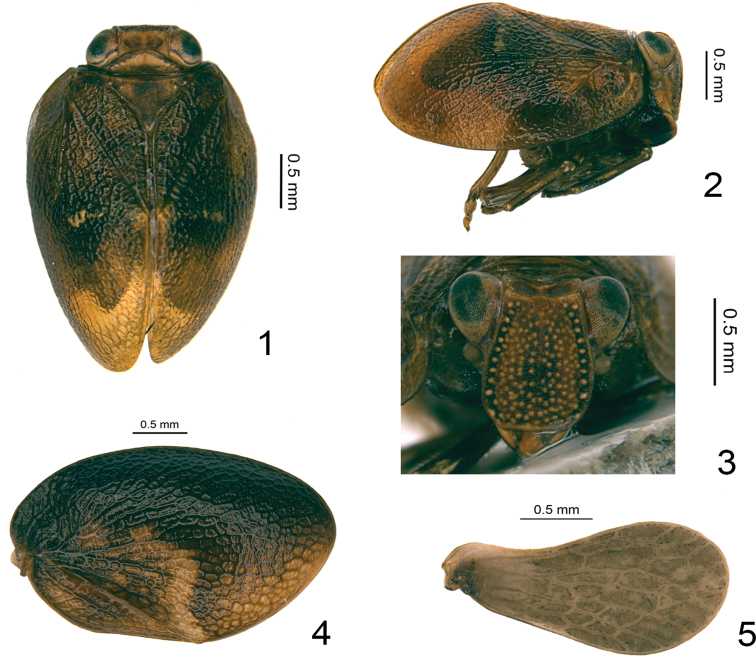
*Retaldar yanitubus* sp. nov. **1** adult (holotype, male), dorsal view **2** adult (holotype, male), lateral view **3** adult (holotype, male), frontal view **4** forewing (paratype, female) **5** hindwing (paratype, female).

This new genus is similar to the genus *Clypeosmilus* Gnezdilov & Soulier-Perkins, 2017 in general appearance, but differs by: 1) a more complex and obscure reticular venation of the forewing (Fig. 1) while a simpler pattern is found in *Clypeosmilus* (Gnezdilov and Soulier-Perkins 2017, fig. 1A); 2) an asymmetrical male genitalia with the periandrium more developed on right side and the aedeagus processes emerging at different levels, more posteriorly on right side (Figs 9, 10) while it is symmetrical in *Clypeosmilus* (Gnezdilov and Soulier-Perkins 2017, fig. 3A); 3) gonostyli with ventral margin deeply convex (Fig. 8), while it is much more elongated in *Clypeosmilus* (Gnezdilov and Soulier-Perkins 2017, fig. 3C). The new genus is also similar to the genus *Eusudasina* Yang, 1994, from which it differs also by its more complex reticulate venation and by its longer frons, around 1.2 times longer (in middle) than broad at widest part (Figs 3, 21), only around 0.9 times longer in *Eusudasina* (Chan and Yang 1994, fig. 34C). With *Euxaldar* Fennah, 1978, *Retaldar* gen. nov. shares the general form of the gonostyli, which is strongly developed ventrally (Fennah 1978, fig. 251; Gnezdilov et al. 2017, fig. 8) but definitively differs by its distal postero-ventral protuberance (Fig. 8) and by its near-symmetric subapical processes on the periandrium (Figs 9, 10) while they are asymmetrical in the former (Gnezdilov et al. 2017, figs 1, 2).

**Figures 6–11.**
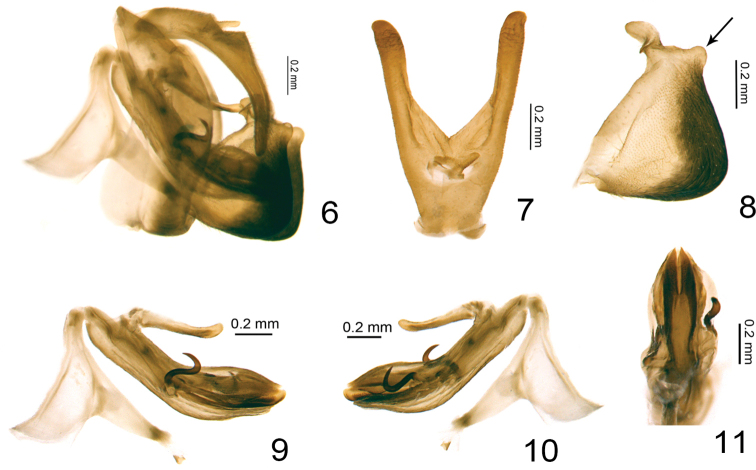
*Retaldar yanitubus* sp. nov., holotype. **6** male genitalia, lateral view **7** male anal tube, dorsal view **8** gonostylus, lateral view **9** phallic complex, left lateral view **10** phallic complex, right lateral view **11** apical half of phallic complex, ventral view. The arrows on Fig. 8 indicates the distal postero-ventral protuberance of gonostyli.

###### Description.

Head with compound eyes slightly wider than pronotum, almost same width as mesonotum (Fig. 1). Vertex rectangular, obviously broader than long at midline, anterior margin almost straight, lateral margins nearly parallel, posterior margin slightly roundly concave at middle; median carina absent on disc (Fig. 1). Frons obviously longer than wide, gradually broadening from dorsal margin to below the level of antennae, then curved to frontoclypeal suture (Figs 3, 21); dorsal margin slightly concave, lateral margins slightly broaden below level of compound eyes, median carina nearly invisible. Frons with numerous tiny tubercles on the whole disc. The tubercles larger on the lateral areas, arranged into a vertical line on each side of frons (Figs 3, 21). Frontoclypeal suture straight (Figs 3, 21). Gena in lateral view flattened and oblique (Fig. 2). Clypeus in lateral view with a protuberance below frontoclypeal suture slightly surpassing the gena (Fig. 2); in ventral view, clypeus without median carina (Figs 3, 21). Rostrum reaching midcoxae; apical segment slightly shorter than subapical one. Antennae with scape extremely short, pedicel rounded (Figs 3, 21). Pronotum triangular, apical margin roundly convex, posterior margin nearly straight, with some faint small nodules on each side or nodules invisible, median carina absent (Figs 1, 19, 20). Mesonotum triangular, a little longer than pronotum in midline, without carina on the disc; with (Fig. 1) or without (Figs 19, 20) some faint small nodules in lateral part apically. Forewings obviously longer than broad, without hypocostal plate, with elevated irregular reticular venations and venation poorly recognizable, costal margin and posterior margin subparallel, costal margin roundly convex, apical margin straight and oblique inward to posterior margin (Fig. 4), CuP obvious, Pcu and A1 fused exceeding middle of clavus (Figs 1, 4). Hindwing one-lobed. Metatibia with two lateral spines on apical half and seven spines apically.

**Figures 12–18.**
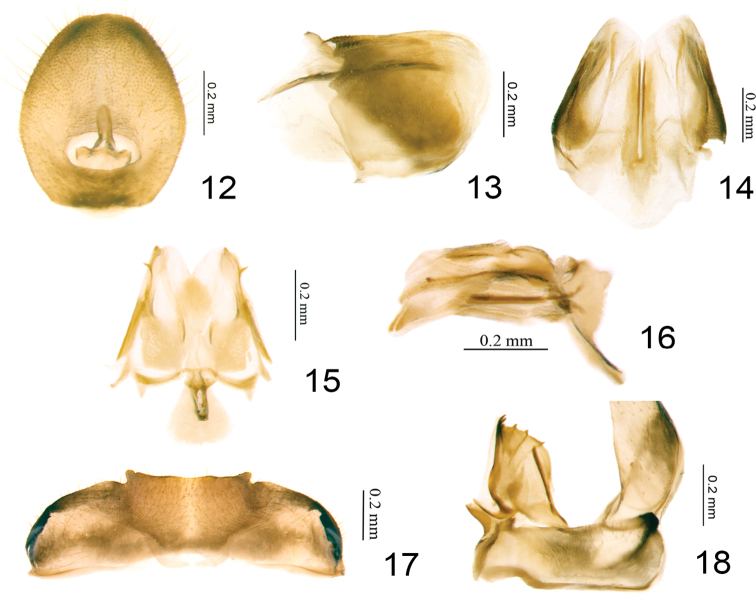
*Retaldar yanitubus* sp. nov., paratype. **12** female anal tube, dorsal view **13** gonoplacs, lateral view **14** gonoplacs, dorsal view **15** gonapophysis IX and gonaspiculum bridge, dorsal view **16** gonapophysis IX and gonaspiculum bridge, lateral view **17** sternite VII **18** gonocoxa VIII and gonapophysis VIII, ventral view.

**Figures 19–21.**
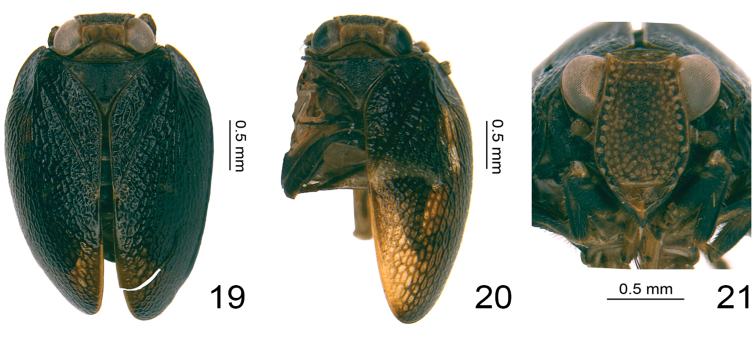
*Retaldar yanitubus* sp. nov., paratype. **19** adult (female), dorsal view **20** adult (female), dorsal view **21** adult (female), frontal view.

###### Male genitalia.

Anal tube in lateral view long and curved (Fig. 6). Pygofer long triangular in lateral view, posterior margin roundly convex (Fig. 6). Gonostyli irregularly quadrangular in profile, almost as high as long, dorsal margin nearly straight, ventral margin strongly convex with a strong posteroapical protuberance, caudo-ventral angle rounded (Figs 6, 8). Capitulum of gonostyli finger-shaped, with a small peaked spine (Fig. 8). Periandrium tubular, subapical nearly symmetric, medially constricted and slightly asymmetric in ventral view. Aedeagus processes asymmetric, right one emerging more posteriorly than left one (Figs 9, 10). Aedeagus a little longer than dorsolateral lobe and ventral lobe of periandrium (Figs 9, 10).

###### Female genitalia.

Anal tube in dorsal view a little longer than wide (Fig. 12). Gonoplacs nearly rectangular in lateral view, apical margin rounded (Fig. 13), in dorsal view fused at middle near base, broadest near base, outer lateral margins straight and roundly convex at base (Fig. 14). Gonapophysis IX in lateral view long and narrow, boat-shaped (Fig. 16); in dorsal view nearly triangular, basal half broader than apical half, with a spine on each side (Fig. 15); gonospiculum bridge developed (Fig. 16). Gonocoxa VIII long rectangular (Fig. 18). Three teeth at apex and three keeled teeth on outer lateral margin of anterior connective lamina of gonapophysis VIII (Fig. 18). Endogonocoxal process membranous (Fig. 18).

###### Distribution.

China (Guangxi).

##### Retaldar yanitubus sp. nov.

Taxon classificationAnimaliaHemipteraIssidae

EDF1FBE6-D0D4-51B8-989C-75E939119C03

http://zoobank.org/7098817F-FAE5-4A30-AE8A-3D4E7DAE2EA1

###### Etymology.

Specific epithet built by the arbitrary combination of the alphabet letter “Y” and “anal tube” latinised into “*anitubus*”, referring to the Y-shaped male anal tube in dorsal view.

###### Diagnosis.

The species is close to *Clypeosmilus centrodasus* Gnezdilov & Soulier-Perkins, 2017, from which it differs by its generic characters (complex reticular venation (Fig. 1), more or less quadrangular gonostyli bearing a postero-apical protuberance) and the form of the male anal tube, which is deeply concave on the apical margin, Y-shaped in dorsal view (Fig. 7), while very shallowly concave and cylindrical in the latter species (Gnezdilov and Soulier-Perkins 2017, fig. 3E). From *Euxaldar guangxiensis* Zhang, Chang & Chen, 2018, another Guangxi species, it differs by: 1) its tegmina pattern black (Fig. 19) to dark tawny with a yellow slender or broader transverse marking (Figs 1, 4, 20), while it is dark brown with more than four yellow irregular markings in *E. guangxiensis* (Zhang et al. 2018, figs 1, 3); 2) the form of the male anal tube which is obviously protruded in the later (Zhang et al. 2018, fig. 10), and 3) the base of periandrium with a finger-shaped dorsal process (Figs 9, 10), while it is with three dorsal processes in *E. guangxiensis* (Zhang et al. 2018, figs 15a, b, 16a, b).

###### Type materials.

**Holotype**: ♂, CHINA: Guangxi Province, Jinxiu, Dayaoshan natural reserve, Hekou, 24°14'11"N, 110°14'11"E, 689.9 m, 23 vii 2018, Feilong Yang & Kun Zhao leg.

**Paratypes**: 2♀♀, same data as for holotype.

###### Description.

Length: male (including forewings) (*N* =1): 3.1 mm; female (including forewings) (*N* =2): 3.3–3.4 mm.

###### Coloration

Vertex tawny, disc with two dark brown circular markings; anterior, lateral and posterior margins tawny (Figs 1, 19, 20). Center of the compound eyes mostly black, surrounded by brown (Figs 1, 3, 20) or compound eyes grayish (Figs 19, 21). Frons mostly tawny, scattered with many yellow nodules on the whole disc (Figs 3, 21); the central area slightly black (Figs 3, 21); lateral areas black, with the yellow nodules arranged into a distinct line on each side (Figs 3, 21); lateral margins tawny (Figs 3, 21). Clypeus in frontal view tawny, with two vertical dark brown markings at middle (Fig. 3) or tawny but the vertical dark brown markings invisible; the basal part black (Fig. 21). Rostrum tawny (Fig. 21). In lateral view gena tawny (Fig. 2), clypeus with a broad black oblique patch covering the base of the gena and most part of the clypeus (Fig. 2). Antennae dark brown (Figs 3, 21). Pronotum tawny, with three small yellow nodules present on each side (Fig. 1) or without them (Figs 19, 20), anterior and posterior margins brown (Figs 1, 19, 20). Mesonotum mainly tawny mixed with some black, disc with two small yellow nodules on each side (Fig. 1) or disc black with nodules almost invisible (Figs 19, 20); anterior margin tawny. Forewings dark tawny, with a discontinuous yellow transverse band from the end of clavus to the middle of forewing (Figs 1, 2) or the transverse marking lighter and broader (Figs 4, 20), or the whole forewing black without any markings (Fig. 19); venations tawny or black, reticular and inconspicuous (Figs 1, 2, 4, 19, 20). Hindwing grayish-brown, with grayish reticulate venations (Fig. 5).

***Head and thorax.*** Vertex 2.5 times wider than long in midline, without median carina; anterior margin straight; posterior margin roundly concaved (Fig. 1). Frons 1.2 times longer in middle than broad at widest part, 1.4 times wider at the widest part than apical margin (Fig. 3). Pronotum with posterior margin 3.6 times wider than long in midline, anterior margin roundly protruded (Fig. 1). Mesonotum with anterior margin 2.0 times wider than long in midline, anterior margin straight (Fig. 1). Forewings 1.6 times longer in longest part than widest part (Fig. 4), clavus obvious, the tip reaching to the middle of forewing in dorsal view (Fig. 1). Metatibiotarsal formula: 2–7/7/2.

***Male genitalia.*** Anal tube in lateral view arc-shaped, gradually narrowing from the base to the end, apical part conical (Fig. 6); in dorsal view anal tube Y-shaped with two long straight posterolateral arms, middle part in between deeply concave; in dorsal view arms as long as median part of anal tube (Fig. 7); anal opening located at the basal 1/4 of anal tube, epiproct protruded (Fig. 7). The highest length of pygofer around 3.4 times of the widest length, no basal notch (Fig. 6). Periandrium with a finger-shaped process originated from dorsal margin of base extending to the middle, directed to caudal (Figs 9, 10); dorsolateral lobe of periandrium a little longer than ventral lobe (Figs 9, 10), the ventral lobe in ventral view rounded in apex (Fig. 11). Aedeagus asymmetric, left hooked process emerging at its mid length, S-shaped, curved and directed dorso-cephalad in lateral view, the tip not exceeding the base of right process (Figs 9, 10); right hooked process almost same length as the left but emerging from its apical 1/4, curved and directed dorsad (Figs 9, 10); apex of aedeagus rounded in lateral view, slightly exceeding dorsolateral and ventral lobe of periandrium (Figs 9, 10). The connective with strongly developed tectiductus (Figs 9, 10).

***Female genitalia.*** Anal tube in dorsal view ovate, widest near middle, 1.2 times longer in midline than widest part, apical margin and lateral margins rounded (Fig. 12); anal opening situated at basal 1/4 (Fig. 12). Gonoplacs in dorsal view fused at middle near base, broadest near base, outer lateral margins straight and roundly convex at base (Fig. 14). Posterior connective lamina of gonapophysis IX in dorsal view nearly triangular, the basal half relatively sclerotized, slightly broader than apical half, apical half membranous, the inner bifurcation at apical 1/3 (Fig. 15); the posterior fibula sclerotized, with a spine on each side on apical 1/4 (Fig. 15). In lateral view, gonapophysis IX long and narrow, dorsal and ventral margins nearly parallel each to another, tip pointed (Fig. 16); gonospiculum bridge in lateral view triangular, needle-like ventrally (Fig. 16). Anterior connective lamina of gonapophysis VIII subrectangular, with three closely situated teeth at apex and three keeled teeth on the outer lateral margin, inner lateral margin without teeth (Fig. 18). Endogonocoxal process membranous, reaching the level of the apical teeth (Fig. 18). Gonocoxa VIII long rectangular, vertical with the gonapophysis VIII (Fig. 18). Hind margin of sternite VII with middle part slightly prominent and truncates in ventral view (Fig. 17).

###### Molecular data.

Genes sequences were registered in GenBank with accession numbers as following: MN381856 (whole 18S), MN381853 (28S D3–D5), MN381851 (28S D6–D7), MN381857 (COXI), MN332232 (Cytb). The COXI sequence of this species differs respectively by 87 bp (over 601 bp: 14.5%) and 103 bp (over 681 bp: 15.1%) from *Eusudasina nantouensis* Yang, 1994 (Genbank accession number: HM052838) and *Clypeosmilus centrodasus* (Genbank accession number: KX761470).

###### Note.

As in the genus *Euxaldar* (Gnezdilov et al. 2017), color and color-pattern variation on forewing is reported in *R. yanitubus* sp. nov. Forewings might be nearly black (Fig. 19) or dark tawny (Figs 1, 2, 20), while color-patterns on the forewing varies from a light yellowish broad traverse band apically curved upward (Fig. 4) to a much thinner band only visible on the middle of forewing (Figs 1, 2) or even absent (Fig. 19).

##### Phylogenetical analysis

**Figure 22.**
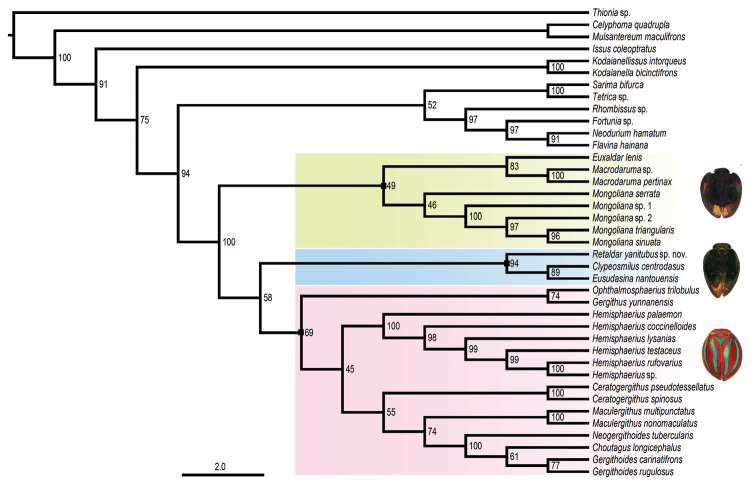
Maximum likelihood tree of Hemisphaeriini based on combined sequences (18S, 28S, COXI, Cytb) with genera of Thioniini, Hysteropterini, Issini, Kodaianellini, Sarimini and Parahiraciini as outgroups. At each node, values denote ML ultrafast bootstrap support

In-group sampling comprised 14 Hemisphaeriini genera and 27 species while 12 other issid genera were used as outgroups (Fig. 22). In all configurations tested, Hemisphaeriini reached a full 100% boostrap (BS). Monophyly of Hemisphaeriina appears less supported (BS=69) than in Wang et al. (2016: BS=98) although the group was recovered in all analyses. Monophyly of Mongolianina is not recovered but appeared paraphyletic, disclosing a new well-supported lineage (BS=94). It includes *Retaldar* gen. nov. at its base in the following relationship (*Retaldar* + (*Clypeosmilus* + *Eusudasina*)). The other Mongolianina taxa form another weakly supported lineage (BS=49) comprising three taxa in the following relationship: (*Mongoliana* + (*Euxaldar*+ *Macrodaruma*)). In several analyses, these two lineages appear in a sister group relationship.

## Discussion

According to their results, Wang et al. (2016) subdivided Issidae into three subfamilies. At their base, Thioniinae Melichar, 1906 with at least two genera, *Thionia* Stål, 1859 and *Cheiloceps* Uhler, 1895 form a monophyletic group sister to the rest of the Issidae. For practical reasons of classification, all New World genera were placed in Thioniinae by Wang et al. (2016), but it is important to note that the subfamily does not necessarily include all of these taxa: *Picumna* Stål, 1864 for instance, never grouped within Thioniinae in their analysis, but in Hemisphaeriinae sec. Wang et al. (2016), although its place is not stabilized. This indicates a mixed origin of New World Issidae, which includes at least an older lineage (*Thionia*, *Cheiloceps*) of Lower Cretaceous origin and another slightly younger one (*Picumna*) originating from the Oriental lineages (Bourgoin et al. 2018). Several other genera are also waiting for a better placement in the issid phylogeny and it might be that other, still younger, taxa have also expanded more recently from Asia into the Nearctic as proposed by Gnezdilov (2019). Only future phylogenetic analyses including New World genera will help to better understand the heterogeneous composition of the Issidae fauna in the Nearctic.

In the phylogeny of Wang et al. (2016), the second subfamily Issinae Spinola, 1839 was less supported (BS = 72). In all subsequent analyses including more taxa (Bourgoin et al. 2018), this node was no more supported, suggesting a paraphyletic Issinae with Issini sec. Wang et al. (2016), no longer sister to Hysteropterini but moving to sister of Hemisphaeriinae as in this study (Fig. 22). Because in some cases Thioniinae also grouped with Hysteropterini, we suggest that these tribes recover separate subfamily ranks in a basal polytomic node with the following topology [Thioniinae, Hysteropterinae, (Issinae + Hemisphaeriinae)].

Within Hemisphaeriinae, Hemisphaeriini sec. Wang et al. (2016) form a natural group of at least 28 genera (Bourgoin 2019) for which monophyly has always been strongly supported in all our analyses since 2016 with a BS=100. Two lineages were recognized as subtribes: Mongolianina and Hemisphaeriina, both well supported (BS = 95 and 98 respectively) in Wang et al. (2016). Addition of new taxa with an almost full set of sequences for the genes under this study has however slightly modified previous results allowing recognition of three main lineages within Hemisphaeriini while eleven other genera still remain in an *incertae sedis* position.

Hemisphaeriina remains the most diverse subtribe with, at least, 11 described genera, eight having being sequenced and providing the following topology: ((*Ophthalmosphaerius + Gergithus*) + (*Hemisphaerius* + (*Ceratogergithus* + (*Maculergithus* + (*Neogergithoides* + (*Choutagu*s + *Gergithoides*)))))). Three genera remain unsequenced: *Epyhemisphaerius* Chan & Yang, 1994, *Neohemisphaerius* Chen, Zhang & Chang, 2014 and *Rotundiforma* Meng, Wang & Qin, 2013. Moreover, *Gergithus yunnanensis* Che, Zhang & Wang, 2007 probably belongs to another new genus and *Gergithus* s.s. should remain in an *incertae sedis* position within Hemisphaeriini. According to Gnezdilov (2017), the *Maculergithus* clade might include two different genera.

According to our analysis, Mongolianina should be now restricted to the following genera: (*Mongoliana* + (*Euxaldar* + *Macrodaruma*)), separated from a third new lineage (*Retaldar* + (*Clypeosmilus* + *Eusudasina*)). The support of this last lineage is high (BS= 94) and higher than the Mongolianina lineage itself. Because the monophyly of the Mongolianina lineage itself remains weak and unstable, we prefer to keep provisionally a paraphyletic Mongolianina, including in it this new lineage. When more genera will be sequenced, a better and stronger topology will probably appear. Genera such as *Bruneastrum* Gnezdilov, 2015, *Tapirissus* Gnezdilov, 2014 and *Neotapirissus* Meng & Wang, 2016 should also join this group, but, as for Hemisphaeriina, new morphological analyses are still needed to better identify these lineages without molecular sequencing.

The new species *Retaldar yanitubus* gen. & sp. nov. has a protruded clypeus in lateral view. In the tribe Hemisphaeriini, several other genera also display such a protruded clypeus: *Euxaldar*, *Clypeosmilus* and *Eusudasina*. They also share a plesiomorphic clearly visible CuP on the forewing while this vein is not visible in Hemisphaeriina(Gnezdilov et al. 2017; Gnezdilov and Soulier-Perkins 2017). Although appearing in two separate lineages, *Retaldar* gen. nov. and *Euxaldar* share also an obscure reticulate forewing venation with the main veins poorly recognizable, while these are clearer in *Clypeosmilus* and *Eusudasina*.

Including more sequenced taxa in the molecular analysis, revisiting morphological characteristics of Hemisphaeriini and investigating the etho-ecology of these fascinating ladybug-like planthoppers is now urgently needed.

## Supplementary Material

XML Treatment for Retaldar

XML Treatment for Retaldar yanitubus
